# Liquid Biopsy in the OMICS Era of Tumor Medicine

**Published:** 2018-03-07

**Authors:** Yi Jiang, Denong Wang

**Affiliations:** Tumor Glycomics Laboratory, SRI International Biosciences Division, USA

**Keywords:** Blood group precursor, Cancer stem cells, Carbohydrate microarray, Circulating tumor cells, Circulating tumor DNA, Glycan markers, Glycomics, Liquid biopsy

## Abstract

Liquid biopsy uses noninvasive blood test to assess tumor heterogeneity and evolution in real time. It looks for tumor components in the blood circulation, such as circulating tumor cells (CTCs) and circulating tumor DNA (ctDNA), to provide tumor-specific information. By detecting multiplex tumor biomarkers, including nucleic acids, proteins, carbohydrates, and other tumor-derived substances, liquid biopsy helps with early tumor diagnosis, tumor evolution monitoring, and prognosis prediction. With the development of high-throughput OMICS tools like carbohydrate microarray and high-speed fiber-optic array scanning technology (FAST scan), it is now practical to identify glycan markers of CTCs and cancer stem cells (CSCs), especially those that are cell-surface exposed and readily accessible for immune recognition and targeting. Potential of this class of biomarkers in tumor subtyping and targeted immunotherapy is yet to be explored.

## Introduction

For long time, traditional tissue biopsy has been considered as “golden standard” for tumor diagnosis and assessment. However, clinicians have never been satisfied, because high risk of complications come with highly invasive biopsy procedure, and only limited samples are available, which prevents us from analyzing tumor heterogeneity and real-time monitoring of tumor evolution [1,2]. Questions have been raised: “Can we find a noninvasive test to provide adequate information of tumor?” As the development of new technologies, liquid biopsy comes as a blood test for tumor analysis. It is considered to have tremendous potential to influence the next generation of precision tumor medicine [3]. Compare to tissue biopsy, liquid biopsy provides a new noninvasive modality to assess tumor heterogeneity and evolution in real time. It uses peripheral blood test to look for tumor components, including CTCs, ctDNA, tumor-derived exosome, and tumor-educated blood platelets (TEPs), etc. [4]. CTCs are tumor cells detected in peripheral blood that escape from primary tumors. A subset of CTCs called cancer stem cells (CSCs), are undifferentiated tumor cells with embryonic characteristics and epithelial-to-mesenchymal transition trait. CTCs/CSCs have ability to give rise to new tumors and play key roles in tumor metastasis [5,6]. CtDNA is tumor-derived fragmented DNA in the bloodstream that could reflect the tumor genome. While CTCs and ctDNA are most widely studied in the clinical and research area, relevant molecular information may also be obtained from analyzing RNAs in the form of exosomes and TEPs [7].

Compare to conventional serum-based biomarkers, liquid biopsy offers tumor-specific information from multiple molecular levels. In the clinical situation, the lack of tumor-specific biomarkers has always been a road block to make good use of current serum-based protein biomarkers. For examples, carcinoma antigen-125 (CA-125), carcinoembryonic antigen (CEA), and prostate-specific antigen (PSA) are found in serum of individuals without cancer, and in substantial portion of patients with advanced cancers, they are not elevated [1,3].

Although clinicians have combined these biomarkers with image and pathological parameters, their utility is greatly limited by low specificity. Liquid biopsy study, especially characterization of CTCs/CSCs molecular features, offers the opportunity to obtain tumor information at the levels of nucleic acids, proteins and carbohydrates, etc.. Moreover, it will facilitate the identification of biomarkers that are exclusively expressed on tumor cells, but not on normal blood cells [7,8].

Liquid biopsy utilizes multiple biomarkers to help with early tumor detection that leads to early treatment. Cohen et al introduced the Cancer SEEK test [9], which was a blood test that utilized combined assays for genetic alterations and protein biomarkers, to identify eight common cancer types in relatively early stage and also to localize the organ of origin of these cancers. Also, for patients with risk factors of developing cancer, CTCs-based liquid biopsy study can be used for early cancer screening[8], like early diagnose lung cancer in chronic obstructive pulmonary disease (COPD) patients [10] and detection of hepatocellular cancer in patients with viral hepatitis and cirrhosis [11].

Liquid biopsy also provides information for prognosis and monitors tumor evolution in real time. Clinical guidelines have reached agreement that numeration of baseline CTCs provides significant prognostic information for metastatic cancers of breast, colon and prostate [8]. Real-time monitoring CTCs enumeration and specific ctDNA mutations related to relapse and resistance of anti-cancer treatment can also guide patient management [7,8].

More and more innovative tools of technologies are bringing novel insights into cancer evolution. For CTCs evaluation, the most popular technology CellSearch [12,13] has involved magnetically tagged antibodies against the epithelial cell surface marker EpCAM for CTCs isolation. To achieve a highly pure CTCs population for better isolation and characterization, “CTC-Chip” and further “CTC-iChip” make use of integrated microfluidic technology [3]. Another technology HD-CTC is able to identify CTCs in high definition based on automated digital microscopy (ADM) [14]. For Analysis of ctDNA, the challenge is not isolation but is to detect genetic alterations in such a small fraction of total DNA in the circulation. Digital polymerase chain reaction (PCR)-based technologies have been used to evaluate point mutations of ctDNA [1]. While for whole genome analyses, Personalized Analysis of Rearranged Ends (PARE) and related approaches [15] use genome rearrangements information for detection of tumor biomarkers in the circulation, another genome-wide method called digital karyotyping [16] detects copy-number alterations. Meanwhile, certain structural alterations in gene regions can also be detected by targeted sequencing [3].

Recognition of abnormal glycosylation in virtually every cancer type has raised great interest in exploring the tumor glycome for biomarker discovery [17–20]. A team of tumor glycomics researchers integrated the use of carbohydrate microarrays and FAST scan to explore cell-surface glycan markers of breast tumor CTCs (bCTCs) and targeting antibodies [6,20–22]. First, using carbohydrate microarrays, anti-tumor monoclonal antibodies (mAbs) were scanned against a large panel of carbohydrate antigens to identify those specific for tumor glycan markers. Subsequently, using FAST-scan technology, the identified mAb was applied to monitor CTC expression of corresponding glycan markers in patients with advanced breast cancer (BCA). This approach revealed the glyco-epitope gp^C1^ as a cell-surface marker of bCTCs and breast tumor CSCs (bCSCs). Interestingly, the gp^C1^ marker is a conserved antigenic determinant of human blood group precursors, i.e., the internal domains or core structures of human blood group substances. These structures are generally masked by other sugar moieties, such as the α -L-fucosyl end groups and other sugar residues essential for blood group A, B, H, or Lewis (Le) active side chains [6,23], but become over expressed or surface-exposed by bCTCs and bCSCs that play vital role in tumor metastasis.

Of note, tumor-associated over expression of blood-group-related autoantigens is not limited to BCA [17,18]. Recently, Gao et al reported the natural ligand of a prostate cancer (PCA)-specific mAb F77 is in fact blood-group H [24,25]. Over expression of gp^F77^ in PCA may reflect increased blood-group H expression together with up-regulated expression of branching enzymes. The mAb C1, which recognizes gp^C1^, differs from F77 in glycan-binding specificities and tumor-binding profiles and does not react with blood group H nor the cell surface targets of PC3 [21,23]. These studies demonstrate epithelial tumor expression of blood group substance-related autoantigens and suggest blood group precursor-based “cryptic” molecules may be appropriate targets for immunotherapy of epithelial tumors.

Liquid biopsy study, together with multiplex tumor biomarkers explored by high-throughput Omics tools, is continuously making progress in the “OMICS” era, where tumor heterogeneity analysis, real-time tumor evolution monitoring, and personalized tumor therapy become applicable in research and ultimately in clinical situation. Meanwhile, there are still questions that could potentially guide future research development. First, rare CTCs and ctDNA in the circulation require more sensitive and reliable detection platforms, especially for early tumor screening and diagnosis. For tumors originated from central nervous system, where physical blood-brain barrier prevents adequate CTCs or ctDNA from entering the circulation [1], noninvasive liquid biopsy faces more challenges in detecting and analyzing tumor information; Secondly, the discordance of tumor biomarkers between primary tumor and metastatic sites, and the heterogeneity of CTCs increase the difficulty of selecting the correct anti-cancer treatment. Thus, further investigation on tumor heterogeneity based on single-cell assessment technologies may provide more information to improve personalized medicine. Lastly, based on the findings of tumor glycomics, natural blood group substance-related autoantigens that expressed on epithelial tumors require further characterization and evaluation. Ultimately, by molecular engineering and chemoenzymatic synthesis of the antigens, more progress in the field of tumor vaccine development and targeted immunotherapy is expected.
